# Oxalate-Metabolising Genes of the White-Rot Fungus *Dichomitus squalens* Are Differentially Induced on Wood and at High Proton Concentration

**DOI:** 10.1371/journal.pone.0087959

**Published:** 2014-02-05

**Authors:** Miia R. Mäkelä, Outi-Maaria Sietiö, Ronald P. de Vries, Sari Timonen, Kristiina Hildén

**Affiliations:** 1 Department of Food and Environmental Sciences, Division of Microbiology and Biotechnology, Viikki Biocenter 1, University of Helsinki, Helsinki, Finland; 2 CBS-KNAW Fungal Biodiversity Centre, Utrecht, The Netherlands; University of Wisconsin - Madison, United States of America

## Abstract

Oxalic acid is a prevalent fungal metabolite with versatile roles in growth and nutrition, including degradation of plant biomass. However, the toxicity of oxalic acid makes regulation of its intra- and extracellular concentration crucial. To increase the knowledge of fungal oxalate metabolism, a transcriptional level study on oxalate-catabolising genes was performed with an effective lignin-degrading white-rot fungus *Dichomitus squalens*, which has demonstrated particular abilities in production and degradation of oxalic acid. The expression of oxalic-acid decomposing oxalate decarboxylase (ODC) and formic-acid decomposing formate dehydrogenase (FDH) encoding genes was followed during the growth of *D. squalens* on its natural spruce wood substrate. The effect of high proton concentration on the regulation of the oxalate-catabolising genes was determined after addition of organic acid (oxalic acid) and inorganic acid (hydrochloric acid) to the liquid cultures of *D. squalens*. In order to evaluate the co-expression of oxalate-catabolising and manganese peroxidase (MnP) encoding genes, the expression of one MnP encoding gene, *mnp1*, of *D. squalens* was also surveyed in the solid state and liquid cultures. Sequential action of ODC and FDH encoding genes was detected in the studied cultivations. The *odc1*, *fdh2* and *fdh3* genes of *D. squalens* showed constitutive expression, whereas ODC2 and FHD1 most likely are the main responsible enzymes for detoxification of high concentrations of oxalic and formic acids. The results also confirmed the central role of ODC1 when *D. squalens* grows on coniferous wood. Phylogenetic analysis revealed that fungal ODCs have evolved from at least two gene copies whereas FDHs have a single ancestral gene. As a conclusion, the multiplicity of oxalate-catabolising genes and their differential regulation on wood and in acid-amended cultures of *D. squalens* point to divergent physiological roles for the corresponding enzymes.

## Introduction

Oxalic acid is a common fungal metabolite that is synthesised as a waste compound by tricarboxylic acid cycle in mitochondria and by glyoxylate cycle in glyoxysomes and peroxisomes [1], [2]. Wood-rotting white- and brown-rot fungi produce oxalic acid as the predominant organic acid and secrete it to their growth medium typically in millimolar quantities [3], [4], [5], [6]. Oxalic acid has been recognised as an important compound affecting fungal growth and metabolism (reviewed by [1]). Moreover, accumulating evidence indicates that the fungal degradation and conversion of wood lignin and carbohydrate polymers is promoted by the fungal secretion of oxalic acid [7], [8], [9], [10], [11]. As oxalic acid is a toxic compound, regulation of its intra- and extracellular concentration is crucial. To achieve this fungi express specific oxalate-degrading enzymes [3], [12], [13], [14].

Wood-rotting basidiomycetes have been shown to produce two types of oxalate-degrading enzymes: oxalate decarboxylases (ODC, oxalate carboxy-lyase, EC 4.1.1.2) and oxalate oxidases (OXO, EC 1.2.3.4). ODC, described from bacteria and fungi, catalyses the conversion of oxalic acid (as oxalate) to formic acid (formate) and CO_2_. ODCs are members of the cupin protein superfamily and belong to the bicupin subclass since they contain two Mn^2+^-binding cupin motifs [15], [16]. OXO is a monocupin enzyme that is evolutionarily related to ODCs and catalyses the cleavage of oxalic acid into two molecules of CO_2_ and H_2_O_2_ [17]. OXO is primarily produced by plants and so far only two white-rot fungi with OXO activity have been reported [13], [18].

In contrast, ODC is prevalent in fungi with often multiple gene copies in one species [19], and several wood-rotting fungi have been reported to produce ODC [3], [12], [14], [20], [21], [22], [23]. The reason for this gene multiplicity is not known, but could point to differences in substrate specificity, kinetic properties or regulation. In wood-rotting fungi, ODC has been proposed to work in conjunction with a formate-degrading enzyme formate dehydrogenase [20], [24]. NAD-dependent formate dehydrogenase (FDH, EC 1.2.1.2) is an intracellular enzyme, which decomposes formate, the reaction product of ODC, resulting in the formation of CO_2_ and NADH. The rapid breakdown of formate is needed to prevent the inhibition of cytochrome oxidase, which is the terminal electron acceptor of the electron transport chain in mitochondria [25]. FDHs are produced by bacteria, fungi and plants [26], but so far only one white-rot fungal FDH protein and two *fdh* genes have been characterized from *Ceriporiopsis subvermispora* [24], [27].

In addition to ODC and FDH, also lignin-modifying heme peroxidases, e.g. manganese peroxidases (MnPs, EC 1.11.1.13), which are indispensable to white-rot fungal wood and lignin decay [19], have been suggested to participate in the breakdown of oxalate [3], [9], [28], [29]. Lignin-modifying peroxidases are considered to degrade oxalate into H_2_O_2_, which is a primary catalyst (oxidant) needed for the catalytic cycle of peroxidases, and therefore enhances peroxidase activity.

Fungal ODCs are suggested to be mainly intracellular enzymes but low ODC activities have been detected also from culture media and from fungal cell wall and extracellular polysaccharide layer [14], [22], [30]. It has been proposed that the principal role of fungal ODCs is to control the intracellular concentration and secretion of oxalic acid as well as to maintain a constant pH and level of oxalate anions outside the hyphae [3], [21]. More recently, ODC and FDH have been proposed to have a broader physiological role than the detoxification. ODC has been suggested to participate in energy production by acting sequentially with FDH inside the fungal cells. The resulting NADH could then be used for ATP synthesis during fungal vegetative growth [24] analogously with a corresponding energy-producing mechanism in methanol-utilizing yeasts [26]. This hypothesis is supported by results from the brown-rot fungus *Postia placenta* that showed simultaneous upregulation of one putative *odc* and three putative *fdh* genes in cellulose medium [20]. Although the physiological role of ODC is still ambiguous, the enzyme has received an increasing interest for use in several biotechnological applications including diagnostics, therapeutics, agriculture and process industry [31].

*Dichomitus squalens* is an efficient lignin-degrading white-rot fungus that grows mainly on conifer trees and decomposes lignin prior to cellulose [32], [33]. We have previously shown that *D. squalens* actively secretes oxalic acid during growth on Norway spruce (*Picea abies*) and in liquid cultures [3]. In addition, the fungus produces high levels of mycelial ODC activity, which is inducible after the supplementation of cultures with excess oxalic acid. We have also cloned and characterized one *odc* gene of *D. squalens* (GenBank ID: FM946037), here designated as *odc1*, and demonstrated its expression during growth on natural wood substrate and in submerged liquid cultures [14].

In order to enlighten the roles of the multiple oxalic-acid catabolising enzymes of *D. squalens*, the expression of the ODC and FDH encoding genes was studied when the fungus was grown on its natural substrate, i.e. spruce wood. The effect of high proton concentration on transcriptional level regulation of the *odc* and *fdh* genes was determined by the amendment of organic acid (oxalic acid) and inorganic acid (HCl) to the liquid cultivations of *D. squalens*. Additionally, the expression of *mnp1* gene of *D. squalens* was followed to evaluate the relevance of lignin-modifying peroxidases in oxalate catabolism. These data imply distinct biological relevance for the individual oxalate-catabolising enzymes of the fungus. Furthermore, insight into the evolution of fungal oxalate-catabolising enzymes showing notable dissimilarities is presented.

## Materials and Methods

### Fungal Cultivations

*Dichomitus squalens* FBCC312 was obtained from the Fungal Biotechnology Culture Collection (fbcc@helsinki.fi), University of Helsinki, Finland, and maintained on 2% (w/v) malt agar plates. The fungus was cultivated as submerged stationary cultures in 70 ml 2% (w/v) liquid malt extract (Biokar) medium and as solid state cultures on 2 g (dry weight) Norway spruce (*Picea abies*) wood sticks at 28°C as described previously [34]. After 7 days of incubation, the malt extract cultures were supplemented with oxalic acid (Sigma-Aldrich) or hydrogen chloride (HCl; Sigma-Aldrich) to a final concentration of 5 µmol/g, and fungal mycelia were harvested after 1 hour and 1 day of induction with the acids.

### Detection of Organic Acids

The pH values and the level of extracellular oxalic and formic acid were determined from three biological replicate malt extract culture liquids of *D. squalens* after 1-h and 24-hour exposure to oxalic acid and HCl. For the extraction of intracellular and mycelial bound oxalic and formic acid, fungal mycelia were filtered through Miracloth (Calbiochem), washed with deionized water and the mycelial dry weight was determined. The mycelia were ground with mortar and pestle under liquid N_2_ and extracted with 0.15% H_3_PO_4_ under agitation on a magnetic stirrer at room temperature for 1 h. The mycelial suspensions were centrifuged at 3 000×g for 10 min at 4°C and the supernatant was collected and used for the quantification of organic acids.

The concentration of intra- and extracellular oxalic and formic acids was detected by high performance liquid chromatography (HPLC) under isocratic conditions using Agilent 1290 Infinity (Agilent Technologies) apparatus equipped with UV-VIS diode array detector. The column, Phenomenex Synergi 4 u Hydro-RP 80A 250×4.6 mm, was maintained at 65°C, and the eluent was 0.15% (v/v) H_3_PO_4_. Three replicate measurements were conducted with each biological replicate. Oxalic and formic acids were used as external standards for quantification. Detection wavelength was 210 nm and reference wavelength was 360 nm.

Differences between the concentration of acids after 1 h and 24 h incubation were determined using the paired-samples *t*-test and *P* values of ≤0.05 were considered to be significant.

### Oxalate Decarboxylase and Formate Dehydrogenase Gene Annotation

Identification of oxalate decarboxylase (ODC) and formate dehydrogenase (FDH) encoding genes in selected fungal genomes from Agaricomycotina species was performed using the search facilities in JGI MycoCosm web portal (http://genome.jgi.doe.gov/programs/fungi/index.jsf). The characterized translated *odc*, *oxo* and *fdh* genes of *D. squalens*, *Trametes versicolor* and *C. subvermispora* were used as a query in a BLASTP analysis and keywords oxalate decarboxylase and formate dehydrogenase as search terms against the selected genomes. Fungal ODC, OXO and FDH amino acid sequences were also retrieved from NCBI database (http://www.ncbi.nlm.nih.gov/). Selected protein models were confirmed with the BLASTP search algorithm (http://www.ncbi.nlm.nih.gov/) and by in silica characterization of the conserved domains (cupins for ODC and D-isomer specific 2-hydroxyacid dehydrogenase catalytic and NAD-binding domains for FDH) using the Pfam database (http://pfam.sanger.ac.uk/search/sequence). Furthermore, the retrieved *D. squalens odc* and *fdh* gene models were manually curated in this work.

The putative transcription factor binding sites were analysed from the 5′ untranslated promoter region of the five *odc* and three *fdh* genes of *D. squalens* using MatInspector 8.0.5 software (http://www.genomatix.de). Nucleotide sequences of 2000 bp in length upstream from the start codons were included in the analyses. The only exception was *fdh1* of which 1753 bp sequence was used.

### Selection of Manganese Peroxidase Encoding Gene from *D. squalens* for Real-time Quantitative Reverse Transcription-PCR (RT-qPCR)

In the white-rot fungus *Ceriporiopsis subvermispora*, three manganese peroxidase (MnP) encoding genes (gene models 50297, 128590, 169968) are expressed during growth on wood [35]. BLAST analysis of the *D. squalens* genome using these three genes as a query all gave the same *D. squalens* gene model (71080) as the closest homolog. The relative expression of this gene, annotated as *mnp1*, was tested in the acid-induced liquid and on solid state spruce cultures and compared to the transcript levels of ODC and FDH encoding genes of *D. squalens*.

### Phylogenetic Analysis

The putative N-terminal signal sequence peptides were removed from the ODC and OXO amino acid sequences according to the prediction with SignalP4.0 program (http://www.cbs.dtu.dk/services/SignalP/) and the putative mature polypeptides were used in the amino acid alignment. Amino acid sequences were aligned using MAFFT [36] and manually corrected in MEGA 5 [37]. Three bacterial ODC-like proteins were used as an outgroup for the phylogenetic tree of fungal ODCs, while three plant FDHs were used as an outgroup for the FDH tree. The phylogeny was computed using three algorithms to provide a high level of confidence. The maximum likelihood (ML) statistical method was performed using the WAG amino acid substitution model [38] with gamma distributed site rates and an invariable site category, as this model provided the best fit to the data. Neighbor joining (NJ) and minimum evolution (ME) trees were computed both using the Poisson model with uniform rates and complete deletion. The stability of the clades was tested with 1000 bootstrap replicates. The ML tree was used as the basis for the displayed trees. For nodes in the ML tree that were also supported by the NJ and ME trees, the bootstraps values of those trees were indicated in the ML tree. A bootstrap value of 50% was used as a cut-off.

### RNA Extraction and cDNA Synthesis

From the submerged liquid cultures, total RNA was extracted from ground mycelia that were frozen with liquid N_2_ as described by Hildén et al. [39]. From the solid state cultures, fungal-colonized spruce sticks were milled under liquid N_2_ with A 11 Basic analytical mill (IKA, Germany) and total RNA was extracted by the method described by Chang et al. [40]. Amount and quality of RNA was determined at 260 nm and 260/280 nm, respectively, by NanoDrop ND-1000D spectrophotometer (Thermo Scientific).

Prior to cDNA synthesis, the RNA samples were tested to be free of DNA contamination by RT-qPCR using the *gapdh* primer pair (see below). Only the RNA batches from which no PCR product was amplified were used as a template in cDNA synthesis.

The cDNA was synthesized with a Smart RACE cDNA Amplification kit (Clontech). The 30 µl reactions contained 130 ng of total RNA, 400 U SuperScript III reverse transcriptase (Invitrogen), 4 µl 5 × first strand buffer, 13 mM dithiothreitol, 0.6 µM 3′ RACE cDNA synthesis primer, 0.6 µM SMART II oligonucleotide and 1.3 mM dNTP mixture (Fermentas). The reactions were conducted according to the manufacturer’s instructions (Clontech).

### Real-time Quantitative Reverse Transcription-PCR (RT-qPCR)

Relative levels of expression of the *D. squalens odc* genes were determined by RT-qPCR. Gene-specific primer pairs for *odc2*, *odc3*, *odc4*, *odc5*, *fdh1*, *fdh2*, *fdh3* and *mnp1* were designed according to the transcript models 163336, 124763, 107932, 100044, 169057, 88541, 51840 and 71080 from the whole genome sequence of *D. squalens* LYAD-421 SS1 (http://genome.jgi-psf.org/Dicsq1/Dicsq1.home.html), respectively, and *odc1* was amplified with a primer pair described by Mäkelä et al. [14]. For *fdh1*, the RT-qPCR primer pair extends to the 5′ untranslated region due to the high similarity (77–81% at coding nucleotide sequence level) between FDH encoding genes of the fungus. The glyceraldehyde-3-phosphate dehydrogenase encoding gene (*gapdh*) was used as an endogenous reference gene to normalize the quantification of *odc*, *fdh* and *mnp1* transcripts in all RT-qPCR reactions [14]. One *gapdh* gene (transcript model 183206) has been annotated in the genome of *D. squalens* LYAD-421 SS1 and its nucleotide sequence is identical with the partial *gapdh* sequence of *D. squalens* FBCC312 (GenBank ID: FM946036). The primer pairs used in this work are listed in Table 1.

**Table 1:** Primers used in RT-qPCR.

Primer	Nucleotide sequence (5′-3′)	cDNA amplicon size (bp)	Reference
*odc1* sense	CTCTTCCCCTCTGGCATTGT	147	[14]
*odc1* antisense	GCCTACGACGAATTCCTTTG		
*odc2* sense	GAGTCAGCTCTACATCTTCC	113	this study
*odc2* antisense	GGACAACCTTCGAGAAC		
*odc3* sense	CGCGGGTGTCGAGTTTCTAC	135	this study
*odc3* antisense	AAGGCGGCTTGGCTGAC		
*odc4* sense	CTGCACTGGCATACATC	133	this study
*odc4* antisense	AGCTAACCGGGAAGAC		
*odc5* sense	TCGCTACCAGGATATCAGC	114	this study
*odc5* antisense	TACTTGGTCTTGCTCAGG		
*fdh1* sense	TCTCCCTGAAATCCCAC	256	this study
*fdh1* antisense	CATTCACGTATGCCGAGTTG		
*fdh2* sense	GACTTGCGCCGCGTACGTA	130	this study
*fdh2* antisense	AGCCAGCTGCGGATACCAAG		
*fdh3* sense	CTGCCCGCTACACGAGGGCA	118	this study
*fdh3* antisense	GACGGCGTCCTTGTCGCAGAT		
*mnp1* antisense	GAGTTCATTCCGCTCAGG	117	this study
*mnp1* antisense	CTCTGCGAGATGGCAATG		
*gapdh* sense	GCTACCGGTGTCTTCACCAC	129	[14]
*gapdh* antisense	TTGACACCGCAGCCAAACAT		

Each of the primer pairs was tested for amplification efficiency by RT-qPCR using six serial dilutions of template cDNA. The PCR efficiency (*E*) was calculated for each primer pair from the slope of the standard curve according to the formula: *E* = [(10^1/slope^) –1] • 100. Amplification efficiencies of the target genes ranged from 90.8% to 109.2% and the efficiency of the reference gene, *gapdh*, was 98.9%.

The cDNA templates were derived from two biological replicates in each time point and treatment, and three technical replicate RT-qPCR reactions were conducted with each cDNA template. A negative control without template was included in each run. The 20 µl RT-qPCR reactions were conducted according to Mäkelä et al. [14] using Maxima SYBR Green qPCR Master Mix (Fermentas) with an ABI 7300 apparatus (Applied Biosystem). Initial denaturation occurred at 95°C for 15 min followed by 35 cycles of (1) denaturation at 94°C for 60 s, (2) annealing at 58°C for 30 s, and (3) elongation at 72°C for 30 s. For melting curve analysis, initial denaturation was performed at 95°C for 15 s, hybridization at 60°C for 30 s, and final denaturation at 95°C for 15 s. Fluorescence was measured during the elongation step.

Results were normalised with *gapdh* and relative expression of the target genes were calculated by the 2^−ΔΔ*C*t^ method [41] and are reported as fold-differences. *odc1* from the non-induced liquid cultures and from the 7 d spruce wood cultures were used as reference (calibrator) samples.

In order to confirm the specificity of RT-qPCR amplification, the dissociation curves were analysed for the presence of a single peak. Furthermore, the amplification products were run on 1% agarose gels, purified with GeneJET Gel Extraction kit (Fermentas) and sequenced (Macrogen Corp., The Netherlands).

Differences between the amount of transcripts in non-induced and in acid-induced cultures were assessed using the paired-samples *t*-test. Repeated measures analysis of variance was performed to test the differences between separate time points of the solid-state wood cultivations. The lognormal distributed ΔC*_t_* values were used in all statistical analyses [42]. *P* values of ≤0.05 were considered to be significant.

## Results

### Oxalate Decarboxylase and Formate Dehydrogenase Genes of *D. squalens*

Analysis of the genome sequence of *D. squalens* LYAD-421 SS1 released at DOE Joint Genome Institute (JGI; http://genome.jgi-psf.org/Dicsq1/Dicsq1.home.html) allowed us to identify five *odc* and three *fdh* gene models in the genome. The length of the genomic sequences of *D. squalens odc* genes varied from 1881 bp to 2539 bp (Table 2). The lowest number of introns, 9, was detected in *odc4* whereas the coding region of *odc1* was interrupted by 17 introns. The amino acid sequence similarity of ODC4 was the lowest (<50%) compared to the other *D. squalens* ODCs, which was supported by the phylogenetical analysis (see: Phylogenetic analysis of oxalate decarboxylases and formate dehydrogenases). The ORFs of *D. squalens odc* genes encoded putative polypeptides of 445–496 amino acids in length (Table 2). All ODCs had a bicupin primary structure and they carried the conserved Mn^2+^ binding residues of three histidines and one glutamate in both of the cupin motifs (Fig. S1A). A putative secretion signal from 19 to 28 amino acids in length was predicted at the N-terminus of all *D. squalens* ODCs (Table 2, Fig. S1A).

**Table 2:** Description of the oxalate decarboxylase (*odc)*, formate dehydrogenase (*fdh*) and manganese peroxidase 1 (*mnp1*) genes and proteins of *D. squalens.*

Gene ID	Protein IDat JGI	GenBank ID	Location in genome	Strand	Protein length (aa)	Signal peptidelength (aa)	gDNA length (bp)	CDS length (bp)	Number of exons
*odc1*	133466	**EJF64740.1**	scaffold_4:889408–894651	–	493	20	2 539	1 479	17
*odc2*	163336	**EJF57579.1**	scaffold_52:66710–68829	–	452	20	1 946	1 356	11
*odc3*	124763	**EJF64520.1**	scaffold_4:204801–206772	+	445	28	1 972	1 317	12
*odc4*	107932	**EJF60197.1**	scaffold_22:382926–384935	–	477	21	1 881	1 431	9
*odc5*	100044	**EJF64733.1**	scaffold_4:863004–866978	+	470	19	2 328	1 410	15
*fdh1*	169057	**EJF62667.1**	scaffold_9:749495–751035	–	386	nd*	1 450	1 160	6
*fdh2*	88541	**EJF60055.1**	scaffold_23:375011–376902	–	381	nd*	1 545	1 146	8
*fdh3*	51840	**EJF65357.1**	scaffold_3:1190689–1192061	–	361	nd*	1 373	1 086	6
*mnp1*	70857	**EJF56747.1**	scaffold_68:93028–94629	–	396	20	1 602	1 191	7

Data is based on *D. squalens* LYAD-421 SS1 genome (http://genome.jgi-psf.org/Dicsq1/Dicsq1.home.html, [19]) and the results of the current study with *D. squalens* FBCC312.

*nd = not detected.

The genomic sequences of *D. squalens fdh* genes were 1373 to 1545 bp in length and interrupted with 6 or 8 introns (Table 2). The three putative FDH polypeptides of *D. squalens* consisted of 361–386 amino acid residues with no predicted N-terminal signal sequences (Table 2). The amino acid residues that are essential for the FDH enzyme catalysis, and substrate and NAD-binding [26] were present in all putative FDHs of *D. squalens* (Fig. S1B). The D-isomer specific 2-hydroxyacid dehydrogenase catalytic and NAD-binding domains were found in all FDH sequences as deduced from Pfam database (http://pfam.sanger.ac.uk/search/sequence).

One putative transcription factor binding site of a pH responsive regulator was detected in the 2000-bp promoter region of *odc1* and *odc3*, located 943 to 959 bp and 1674 to 1690 bp upstream of the start codon, respectively. Two putative pH responsive regulator binding sites, situated at −749 to −765 bp and −1691 to −1707 bp, were found in the promoter region of *odc2* and even three, situated at −1238 to −1254 bp, −1593 to −1609 bp and −1681 to −1697 bp, in the promoter region of *odc5*. No putative pH responsive transcription factor binding sites were found within the studied promoter sequence of *odc4* gene. For the FDH encoding genes, one putative pH responsive transcription factor binding site was detected at 265 to 281 bp upstream of of *fdh1*, and three at 786 to 802 bp, 1462 to 1478 bp and 1578 to 1594 bp upstream of *fdh3* start codon, respectively, whereas no putative binding sites for pH responsive regulators were present in the promoter region of *fdh2*.

### Intra- and Extracellular Oxalic and Formic Acid in Liquid Cultivations

**Figure 1:**
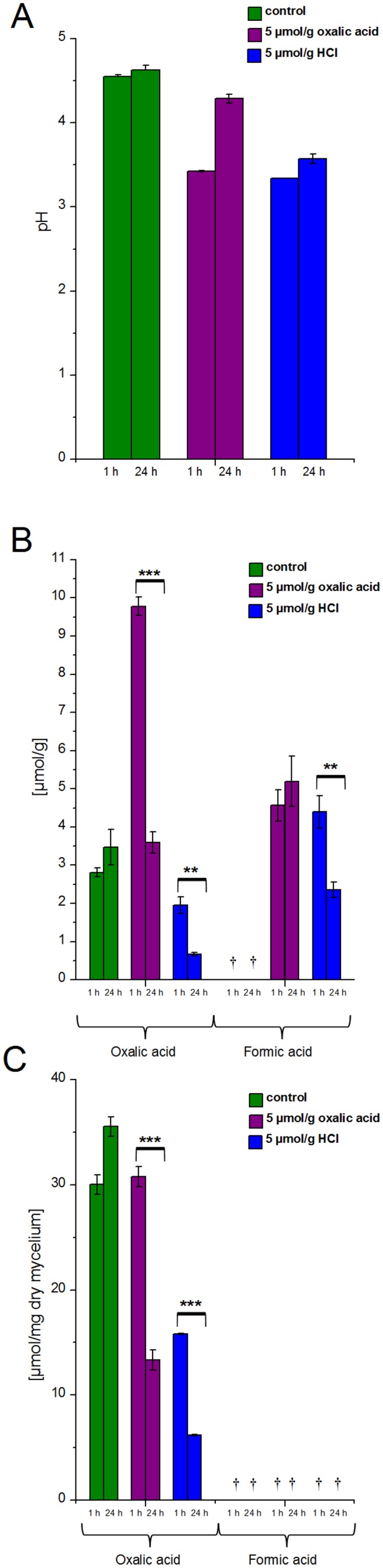
Effect of acid-amendment to the liquid cultures of *D. squalens*. A) Extracellular pH values and B) concentration of extracellular, C) intracellular and mycelial-bound oxalic and formic acid were determined from control cultivations and cultivations added with 5 µmol/g of oxalic acid or HCl after 1 h and 24 h. Error bars represent the standard deviation between three parallel cultivations. ^†^under detection limit; ^**^ *P*≤0.01, ^***^ *P*≤0.001, significant difference between the samples by paired-samples *t*-test.

The supplementation of oxalic acid and HCl acidified the culture liquids of *D. squalens* from pH 4.6 to pH 3.4 and 3.3, respectively (Fig. 1A). In the oxalic-acid amended cultures the fungus increased the extracellular pH value back to the level of the control cultures, i.e. pH 4.3, after 24-hour induction. In the HCl-amended cultures only slight increase, to pH 3.6, was detected.

As indication of induction of ODC activity, formic acid accumulated in the growth liquid of *D. squalens* already after 1 h and was still detected after 24 h exposure to both organic and inorganic acid (Fig. 1B). In oxalic-acid amended cultivations the concentration of formic acid was 4.6 µmol/g and 5.2 µmol/g, and in HCl-amended cultivations 4.4 µmol/g and 2.4 µmol/g after 1 h and 24 h exposure, respectively. In the control cultivations no formic acid was observed. In accordance with the appearance of formic acid, a significant decrease in the extracellular oxalic acid concentration was detected after 24 h in the cultivations that were amended with either 5 µmol/g oxalic acid (2.7-fold decrease, *P*≤0.001) or 5 µmol/g HCl (2.9-fold decrease, *P*≤0.05) (Fig. 1B). Significant decline (*P*≤0.05) in the concentration of formic acid was observed in the HCl-amended cultivations after 24 h, thus possibly pointing to FDH activity.

Consistent with the culture liquids, the level of intracellular and mycelial bound oxalic acid decreased significantly after 24-h induction in both oxalic-acid (2.3-fold decrease, *P*≤0.001) and HCl-amended (2.5-fold decrease, *P*≤0.001) cultivations (Fig. 1C). No formic acid was detected in the mycelial extracts from either control or acid-amended cultivations of *D. squalens*, thus indicating the rapid detoxification of formic acid.

### Transcript Levels in Acid-induced Liquid Cultivations

**Figure 2:**
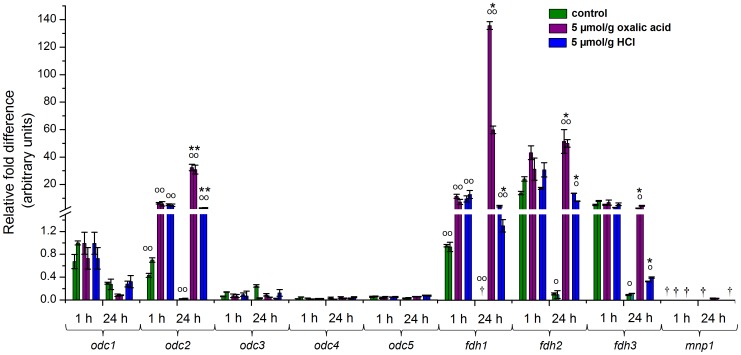
Relative expression of *D. squalens odc*, *fdh* and *mnp1* transcripts in acid-amended liquid cultures. Results are shown for two biological replicate cultures with and without addition of 5 µmol/g oxalic acid or HCl. Error bars represent the standard deviation between three technical replicates. ^†^under detection limit; ^○^ *P*≤0.05, ^○○^ *P*≤0.01, significant difference between non-induced and acid-induced cultures by paired-samples *t*-test; ^*^ *P*≤0.05, ^**^ *P*≤0.01, significant difference between oxalic acid and HCl-amended cultures by paired-samples *t*-test.

The effect of high proton concentration on the relative transcript levels of *odc*, *fhd* and *mnp1* genes was examined by real time RT-qPCR in the submerged malt extract liquid cultivations. The organic acid (oxalic acid) and inorganic acid (HCl) supplemented cultivations were compared to the unexposed control cultivations. A strong upregulation of the expression of *odc2* (*P*≤0.01) was observed already 1 h after the addition of both organic and inorganic acid (11-fold and 9-fold, respectively), and the level of *odc2* transcripts increased further after 24 h induction (Fig. 2). Furthermore, the addition of oxalic acid promoted the expression of *odc2* most, and after 24-h exposure the accumulation of *odc2* transcripts was significantly higher (11-fold, *P*≤0.01) when compared to the HCl-supplemented cultivations. The *odc1* transcripts were the most abundant in the control cultivations without supplementation of excess oxalic acid or HCl (Fig. 2). However, the expression of *odc1* was not observed to change significantly in the acid amended cultures, thus suggesting a constitutive expression of *odc1*. Also, a low constitutive expression level was detected for *odc3*, *odc4* and *odc5* in all studied conditions (Fig. 2).

The expression of all three *fdh* genes was upregulated in both acid-induced liquid cultivations (Fig. 2), which is in accordance with the formic acid detected in the extracellular culture liquids (Fig. 1B). The most rapid response was detected with *fdh1*, which expression was significantly promoted already after 1 h acid exposure (*P*≤0.01), and resulted in a 10- and 12-fold increase in oxalic acid and HCl-amended cultivations, respectively. In addition, the amount of all three *fdh* transcripts increased significantly after 24-h induction when compared to the control cultures (*P*≤0.01 for *fdh1*, *P*≤0.01 and *P*≤0.05 for *fdh2*, and *P*≤0.05 for *fdh3*). Furthermore, significantly higher (*P*≤0.05) expression of *fdh* genes was observed in oxalic-acid than in HCl amended cultures after 24 h (Fig. 2).

The *mnp1* gene was transcribed at low level only in the oxalic-acid amended cultivations after 24-h exposure to the organic acid (Fig. 2).

### Transcript Levels on Solid State Wood Cultivations

**Figure 3:**
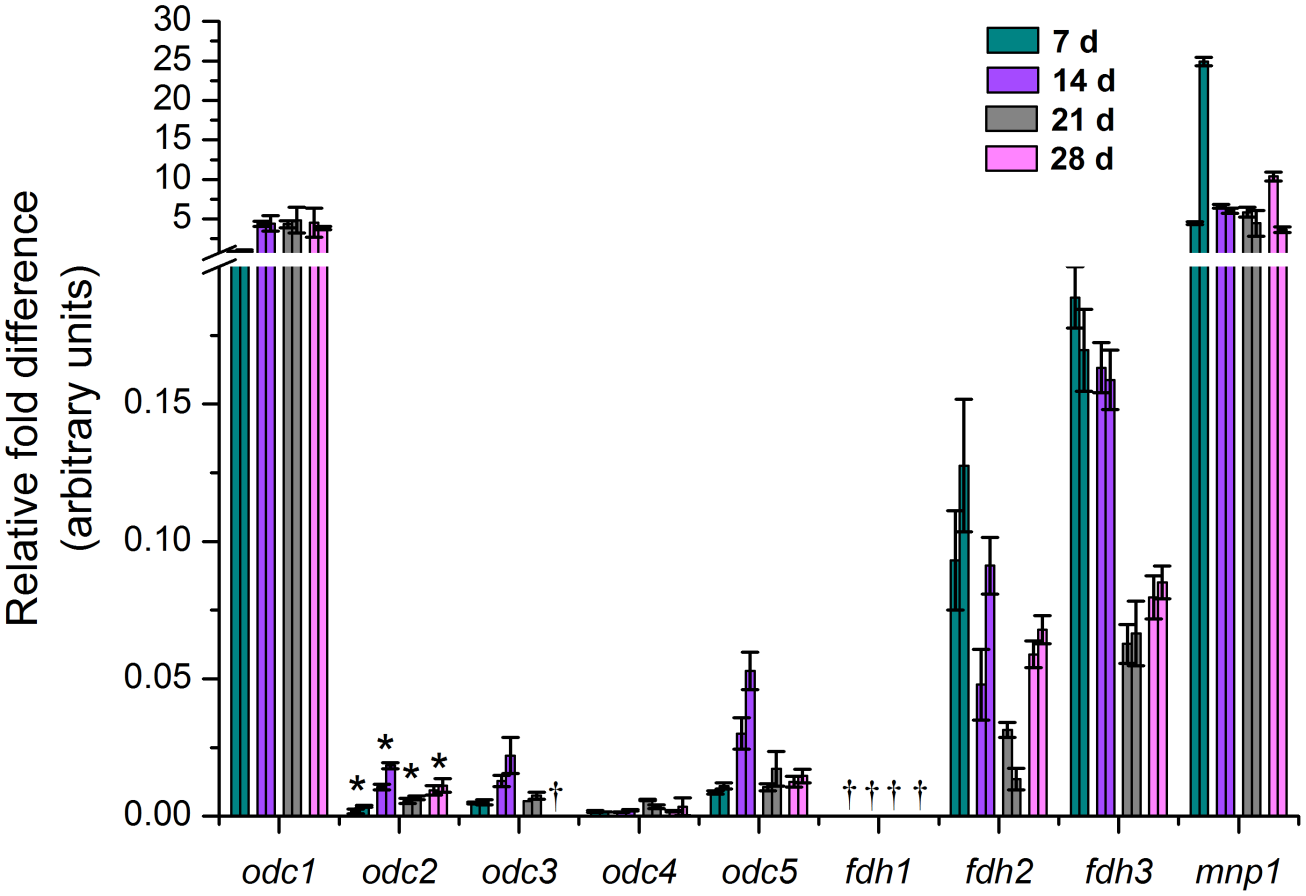
Relative expression of *odc*, *fdh* and *mnp1* transcripts during cultivation of *D. squalens* on spruce wood. Results are shown for two biological replicates. Error bars represent the standard deviation between three technical replicates. ^†^under detection limit; ^*^ *P*≤0.05, significant differences between the time points by repeated measures analysis of variance.

To obtain further insights into oxalic acid metabolism and, more specifically, the transcriptional regulation of oxalate-degrading enzymes during fungal growth on natural lignocellulose, the relative expression levels of *odc*, *fdh* and *mnp1* transcripts were quantified after 1, 2, 3 and 4 weeks of cultivation of *D. squalens* on solid-state spruce wood stick cultures by RT-qPCR. The *odc1* transcript was the most abundant, and in line with the liquid cultivations it showed constitutive expression throughout the cultivation (Fig. 3). The level of the other *odc* transcripts remained very low at all the time points studied, and *odc3* transcripts were not detectable after 4 weeks of cultivation. Only the transcript level of *odc2* changed significantly (*P*≤0.05) during the growth of *D. squalens* on wood thus pointing to transcriptional regulation for this gene (Fig. 3). However, the *odc2* transcripts were detected at 400 to 700-times lower amount than the *odc1* transcripts, which possibly implies a minor role for ODC2 enzyme in the natural growth conditions of the fungus.

From the *fdh* genes, *fdh3* was expressed at the highest level during *D. squalens* cultivation on spruce (Fig. 3). The *fdh3* transcripts were 1 to 5 times more abundant than *fdh2*, whereas the level of *fdh1* transcripts remained under the detection limit throughout the cultivation. The amount of *fdh2* and *fdh3* transcripts showed no statistically significant differences during the cultivation therefore suggesting that these genes are constitutively expressed on spruce.

The *mnp1* was expressed constitutively in the course of the spruce cultivation of *D. squalens* (Fig. 3), and the relative transcript amounts were at comparable level with *odc1*.

### Phylogenetic Analysis of Oxalate Decarboxylases and Formate Dehydrogenases

**Figure 4:**
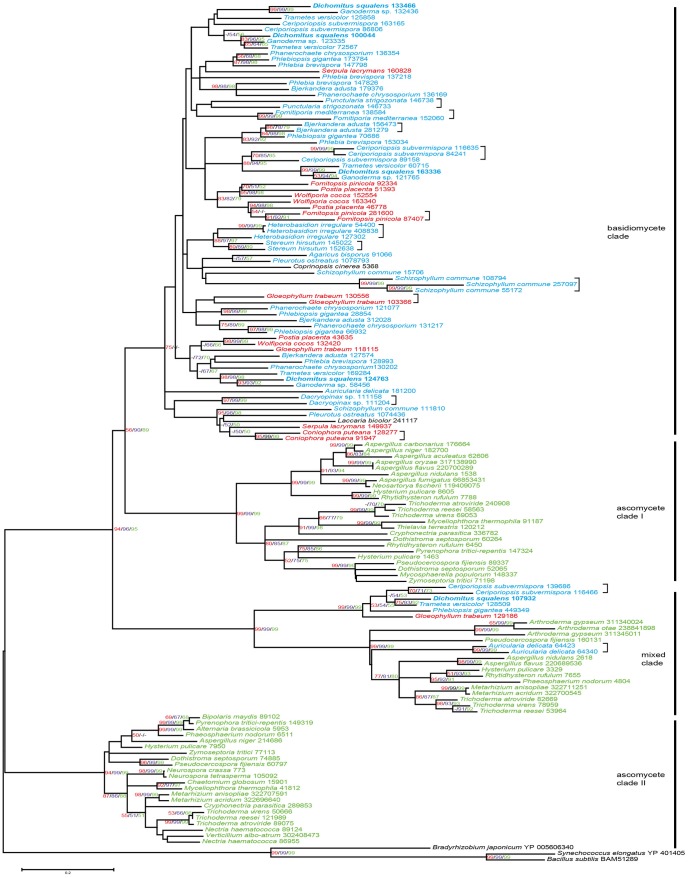
Maximum likelihood (ML) tree of selected fungal ODCs. Three bacterial sequences were used as an outgroup. The scale bar shows a distance equivalent to 0.2 amino acid substitutions per site. Values over 50% bootstrap support are shown with ML values in red, Neigbor Joining values in purple and Minimum Evolution values in green. Putative recent gene duplications are indicated by square brackets. Species names are followed by protein IDs from JGI (http://genome.jgi-psf.org/programs/fungi/index.jsf) or sequence accessions from NCBI database (http://www.ncbi.nlm.nih.gov/protein). Proteins of basidiomycetous white-rot and brown-rot species are in blue and red, respectively, while proteins of other basidiomycetes are in black. Ascomycete proteins are in green. The *D. squalens* proteins are in bold.

The phylogenetic tree of ODCs shows one basidiomycete clade, two ascomycete clades and one clade consisting of a basidiomycete and an ascomycete subclade (Fig. 4). Interestingly, in this combined clade, two putative ODCs from the basidiomyceteous jelly fungus *Auricularia delicata*, causing white-rot in wood, are situated in the ascomycete subclade. Numbers of putative ODCs differ between 0–7 in basidiomycetes and between 0–4 in ascomycetes (Table S1). For basidiomycetes, 14 putative cases of recent gene duplications were observed (Fig. S2), which can be seen by the presence of two genes from a single species clustering together and clearly separate from the nearest other species (Fig. 4). In ascomycetes only a single recent duplication was observed (Fig. S2B).

**Figure 5:**
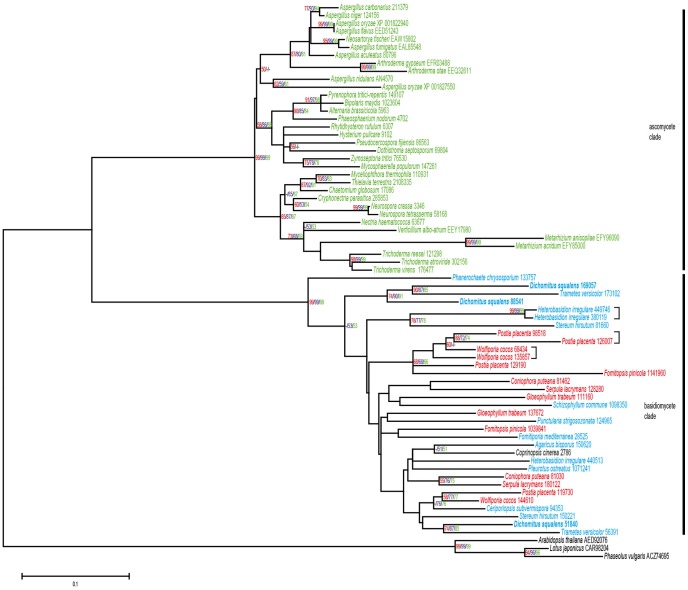
Maximum likelihood (ML) tree of selected fungal FDHs. Three plant sequences were used as an outgroup. Values over 50% bootstrap support are shown with ML values in red, Neigbor Joining values in purple and Minimum Evolution values in green. The scale bar shows a distance equivalent to 0.1 amino acid substitutions per site. Putative recent gene duplications are indicated by square brackets. Species names are followed by protein IDs from JGI (http://genome.jgi-psf.org/programs/fungi/index.jsf) or sequence accessions from NCBI database (http://www.ncbi.nlm.nih.gov/protein). Proteins of basidiomycetous white-rot and brown-rot species are in blue and red, respectively, while proteins of other basidiomycetes are in black. Ascomycete proteins are in green. The *D. squalens* proteins are in bold.

In contrast with the ODC tree, only three and one putative recent duplication events were observed in the phylogenetic tree of FDHs for basidiomycetes and ascomycetes, respectively. In addition, the FDH tree shows a clear division between ascomycete and basidiomycete sequences (Fig. 5). The average number of putative FDH encoding genes differs between 0–4 in basidiomycetes (Table S1). All ascomycetes have only one FDH encoding gene, except *Aspergillus oryzae*, which harbours two putative FDHs.

## Discussion

The current study suggests a pivotal role of ODC1 during the growth of *D. squalens* on coniferous wood. In addition, the constitutive expression of *odc1* on spruce and in acid-induced cultivations corroborates our earlier results of this particular gene [14]. This is also consistent with the transcriptome and proteome studies of a white-rot fungus *Phanerochaete chrysosporium* and a brown-rot fungus *P. placenta* showing the active role of oxalate-degrading enzymes during fungal growth on wood [20], [23], [43], [44].

The significantly induced expression of *odc2* at high proton concentration may demonstrate the importance of ODC2 in extreme environmental conditions with high acidity. Correspondingly, the transcription of an ODC encoding gene of a white-rot fungus *F. velutipes* has been shown to be upregulated by a low pH [45]. This also resembles the induction of a bacterial ODC enzyme from *Bacillus subtilis* (OxdC), which is not inducible by oxalate, but by low pH [46]. A functional low pH-responsive element that specifically binds an acid-inducible transcription factor has been detected within the promoter region of *F. velutipes* ODC encoding gene [45]. Whether the putative transcription factor binding sites for pH responsive regulators present within the promoter region of the *D. squalens odc* genes are functional still needs to be studied.

Differentially regulated expression of two *odc* genes has also been reported for a brown-rot fungus *Fibroporia radiculosa* during growth on copper-treated southern yellow pine sapwood wafers [47]. The genome of *F. radiculosa* harbours 7 putative *odc* gene models in total thus suggesting that the distinct oxalate-degrading genes are differentially regulated and expressed as a response to the changing environmental conditions [47]. In addition, significant variation in the expression of ODC encoding genes caused by different wood species, i.e. aspen (*Populus grandidentata*) and pine (*Pinus strobus*), as a carbon source has been shown for the white-rot fungus *P. chrysosporium* [44].

The rapid disappearance of oxalic acid from the acid-induced liquid cultivations of *D. squalens*, also observed previously [3], is most probably due to the activity of ODC2 protein. It was also shown that *mnp1* is not induced by either organic or inorganic acid amendment. Only a very low transcript level of *mnp1*was detected 24-h after the addition of oxalic acid, and therefore MnP1 probably has minor role in the degradation of oxalic acid in these conditions. Furthermore, strong upregulation of *odc2* transcription seemingly explains our former observation of the promotion of ODC activity in *D. squalens* after addition of 5 mM oxalic acid [14]. Correspondingly, the addition of 5 mM oxalic acid caused a rapid decline in the extracellular oxalic acid concentration in the cultures of a white-rot fungus *Bjerkandera fumosa* in conjunction with the appearance of formic acid [48]. The increase in mycelial ODC activity after addition of inorganic acids including HCl has also been reported for a white-rot fungus *T. versicolor* [49]. In *T. versicolor*, two times higher ODC activity was achieved by the amendment of inorganic acids when compared to oxalic acid, thus suggesting that the high H^+^ concentration would be the causative agent of induction. Our results cannot, however, exclude the specific upregulation of *odc* genes by oxalic acid as the amount of *odc2* transcripts was significantly higher in the oxalic-acid amended than in the HCl-amended cultivations of *D. squalens*. On the other hand, exposure to higher H^+^ concentration (1.3-fold) in HCl-induced cultures may already have caused an inhibitory effect resulting in lower transcript amount of *odc2*.

The low constitutive expression level of *odc3*, *odc4* and *odc5* suggests that the corresponding ODC enzymes had minor roles in the decomposition of oxalic acid in the studied cultivations of *D. squalens*. The significance of those genes in specific growth conditions remains to be elucidated.

Our results indicate that FDH2 and FDH3 work consecutively with ODC1 and are the enzymes responsible for the oxidation of intracellular formate to CO_2_ when *D. squalens* grows on its natural wood substrate. This corroborates the earlier report of sequential action of basidiomycetous ODC and FDH enzymes reported from the white-rot fungus *C. subvermispora* [24]. A physiological connection between ODCs and FDHs has also been proposed in the brown-rot fungus *P. placenta*, in which upregulation of one *odc*, three *fdh*s and one putative formate transporter gene has been shown when the fungus was grown in cellulose medium [20].

However, in the acid-induced liquid cultivations of *D. squalens*, extracellular formic acid accumulated apparently as a result of vigorous ODC activity, resulting in the rapid upregulation of *fdh1* transcription, whereas the two other FDH encoding genes were induced more slowly. Therefore, FDH1 most probably has a role in the degradation of high concentrations of formic acid. In addition, this result supports the earlier study with *C. subvermispora* reporting an increase of FDH activity as a response to formic acid amendment, which was suggested to be principally due to the intense expression of one particular gene, *CsFDH1* [27].

High level of constitutive expression of *mnp1* shows the fundamental role of the corresponding enzyme during the growth of *D. squalens* on spruce wood. The concomitant expression of oxalate-catabolizing and MnP encoding genes reflects the necessity of oxalic acid metabolism and lignin-modifying peroxidase activity in wood lignocellulose decay. It is also possible that, in addition to the specific oxalate-degrading enzymes, lignin-modifying peroxidases also take part in the degradation of oxalic acid during the fungal growth on plant biomass [3], [9], [28], [29].

The evolution of ODCs and FDHs in fungi shows some remarkable differences. There appears to be a single ancestral gene for FDH, since the phylogenetic tree of FDHs is split into two clades, one containing ascomycete sequences and one containing basidiomycete sequences. In contrast, the phylogenetic tree of ODCs contains two ascomycete clades, a basidiomycete clade and a clade containing both ascomycete and basidiomycete sequences. This suggests that the ancestral fungus already contained (at least) two copies of ODCs. Overall, number of ODC encoding gene models is higher in basidiomycetes, thus indicating a broader role for these enzymes in this group of fungi than for ascomycetes. As oxalic acid has been proposed to be involved in decay of plant biomass [11], [29], this may explain the expansion in basidiomycetes. Another striking difference between the FDH and ODC trees is the large number of apparent recent gene duplications of putative ODC encoding genes in basidiomycetes. This was suggested by the presence of two genes of a single species occurring as a separate subclade in the tree. While multiple putative FDH encoding genes are also observed in several basidiomycetes, they are present in separate subbranches in the tree, indicating that these duplications already occurred before the division into the current species. The reason for this high frequency of recent duplication of ODC encoding genes requires further study.

This study shows the genome-wide transcription of all ODC and FDH encoding genes and demonstrates their consecutive action at the transcript level in the white-rot fungus *D. squalens* under different growth conditions. While our results suggest the constitutive metabolic role of *D. squalens* ODC1, FDH2 and FDH3, it is also conceivable that ODC2 and FHD1 are the main responsible enzymes of *D. squalens* for the detoxification of high concentrations of oxalic and formic acids. This can be crucial in nature where high oxalic acid concentrations, secreted e.g. by brown-rot fungi, may inhibit the growth of more sensitive fungi, thus having an impact on competition between fungal species [1]. Gene deletions could confirm these roles, but this requires development of a transformation system for this fungus, which currently does not exist. Based on the results of this study, the oxalate-catabolising genes of *D. squalens* are differentially regulated and show distinct evolution thus indicating that multiplicity of the genes is most probably not due to gene redundancy, but it enables the growth and survival of the fungus in varying environmental conditions.

## Supporting Information

Figure S1 **Alignment of the translated amino acid sequences of** ***D. squalens*** **A) ODCs and B) FDHs by Muscle in Geneious 5.3.6 software package (Geneious 5.3.6 created by Biomatters.** Available from http://www.geneious.com/). A) Putative N-terminal signal peptide sequences are underlined, two cupin motifs are boxed, conserved Mn^2+^ binding amino acid residues are marked with * and the amino acid position corresponding to Glu162 in *Bacillus subtilis* OxdC is marked with ▾. B) The key amino acid residues for the catalysis of FDH and the binding of coenzyme and substrate are boxed.
(PDF)

Figure S2 **Distribution of ODC and FDH encoding genes in the fungal kingdom.** Number of *odc* and *fdh* gene models are in green and pink, respectively. Numbers in brackets are the number of gene models originating from recent gene duplication. A) Basidiomycetes. White-rot species are in blue, brown-rot species are in red. B) Ascomycetes.
(PDF)

Table S1 **List of species used in this study split in ascomycetes and basidiomycetes.** Numbers of putative ODC and FDH encoding gene models are listed as are the number of gene models that have likely originated from recent gene duplication.
(PDF)
