# Supplementary material for: Oxalate-Metabolising Genes of the White-Rot Fungus Dichomitus squalens Are Differentially Induced on Wood and at High Proton Concentration
Source: PLoS One. 2014 Feb 5;9(2):e87959. doi: 10.1371/journal.pone.0087959 (PMC3914892; doi:10.1371/journal.pone.0087959)

Supplementary Figure 1. Alignment of the translated amino acid sequences of *D. squalens* A) ODCs and B) FDHs by Muscle in Geneious 5.3.6 software package (Geneious 5.3.6 created by Biomatters. Available from http://www.geneious.com/). A) Putative N-terminal signal peptide sequences are underlined, two cupin motifs are boxed, conserved Mn^{2+} binding amino acid residues are marked with * and the amino acid position corresponding to Glu162 in *Bacillus subtilis* OxdC is marked with ▼. B) The key amino acid residues for the catalysis of FDH and the binding of coenzyme and substrate are boxed.

A

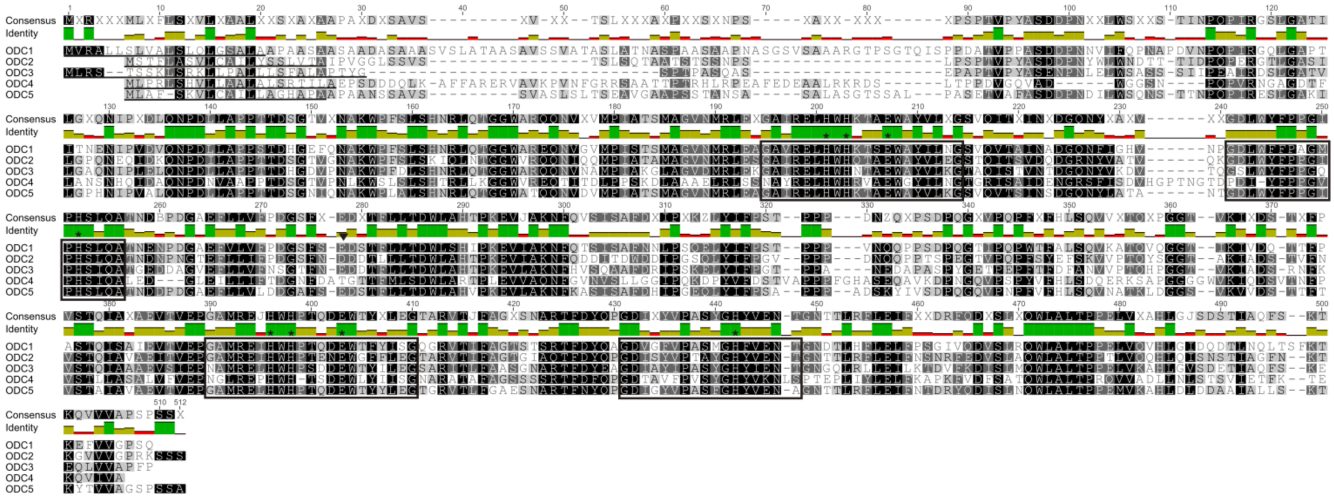

B

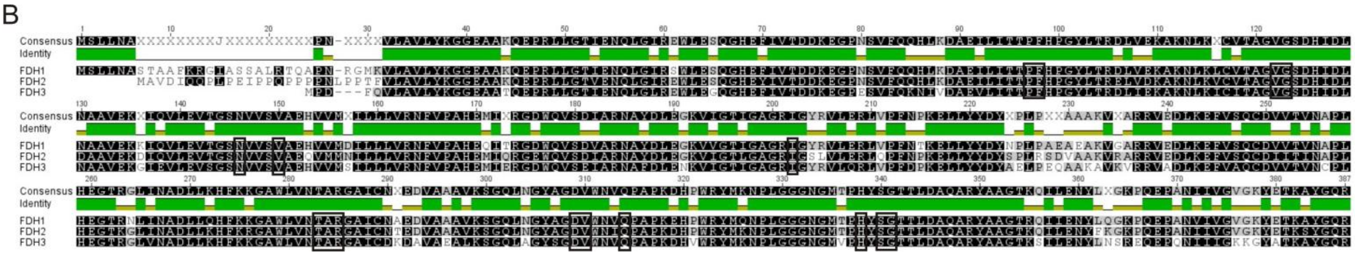

Supplement: Figure S1 — Alignment of the translated amino acid sequences of D. squalens A) ODCs and B) FDHs by Muscle in Geneious 5.3.6 software package (Geneious 5.3.6 created by Biomatters. Available from http://www.geneious.com/). A) Putative N-terminal signal peptide sequences are underlined, two cupin motifs are boxed, conserved Mn2+ binding amino acid residues are marked with * and the amino acid position corresponding to Glu162 in Bacillus subtilis OxdC is marked with ▾. B) The key amino acid residues for the catalysis of FDH and the binding of coenzyme and substrate are boxed. (PDF) [file pone.0087959.s001.pdf]
