# Supplementary material for: Oxalate-Metabolising Genes of the White-Rot Fungus Dichomitus squalens Are Differentially Induced on Wood and at High Proton Concentration
Source: PLoS One. 2014 Feb 5;9(2):e87959. doi: 10.1371/journal.pone.0087959 (PMC3914892; doi:10.1371/journal.pone.0087959)
Supplement: Figure S2 — Distribution of ODC and FDH encoding genes in the fungal kingdom. Number of odc and fdh gene models are in green and pink, respectively. Numbers in brackets are the number of gene models originating from recent gene duplication. A) Basidiomycetes. White-rot species are in blue, brown-rot species are in red. B) Ascomycetes. (PDF) [file pone.0087959.s002.pdf]

Supplementary Figure 2. Distribution of ODC and FDH encoding genes in the fungal kingdom. Number of *odc* and *fdh* gene models are in green and pink, respectively. Numbers in brackets are the number of gene models originating from recent gene duplication. A. Basidiomycetes. White-rot species are in blue, brown-rot species are in red. B. Ascomycetes.

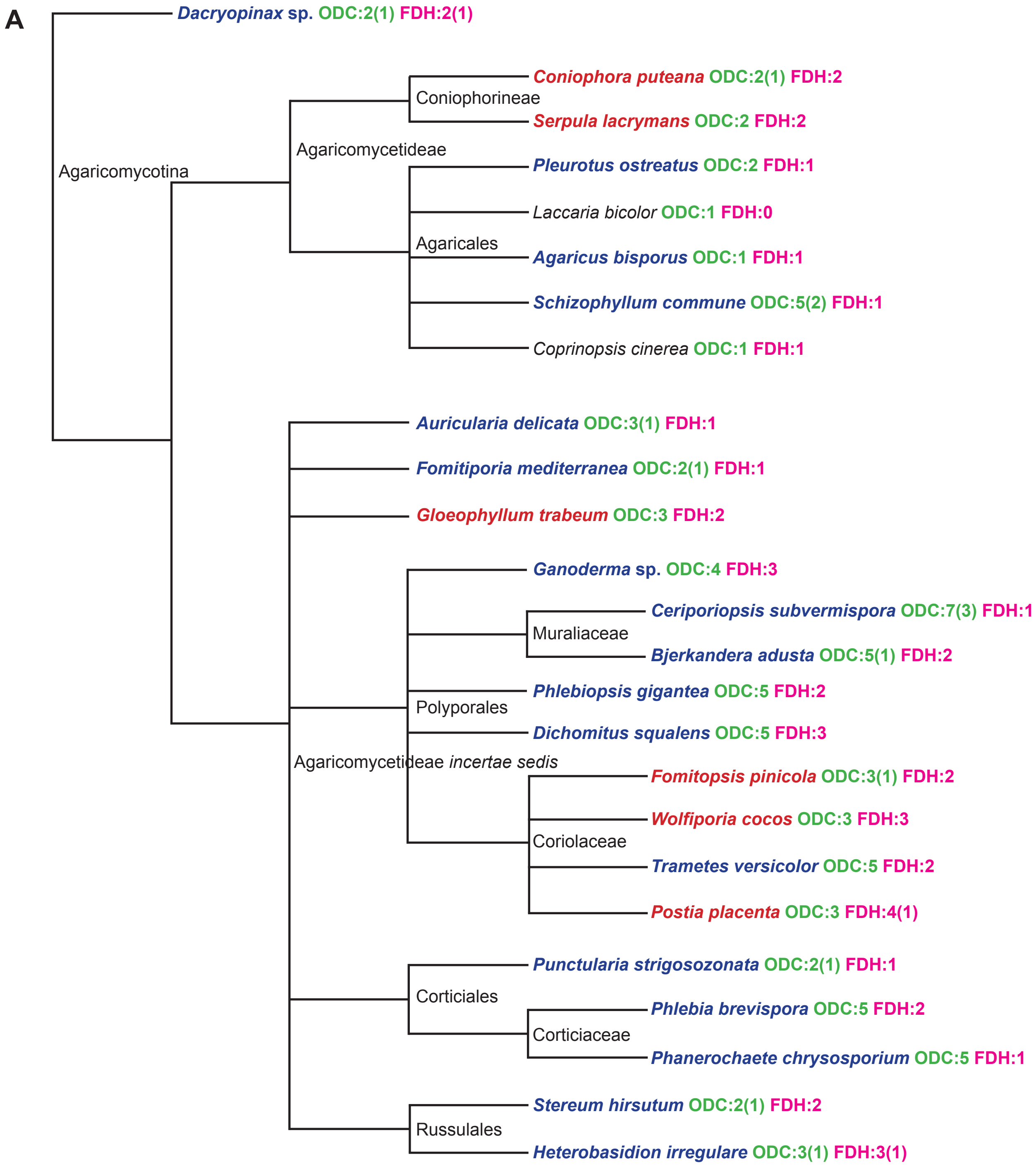

B

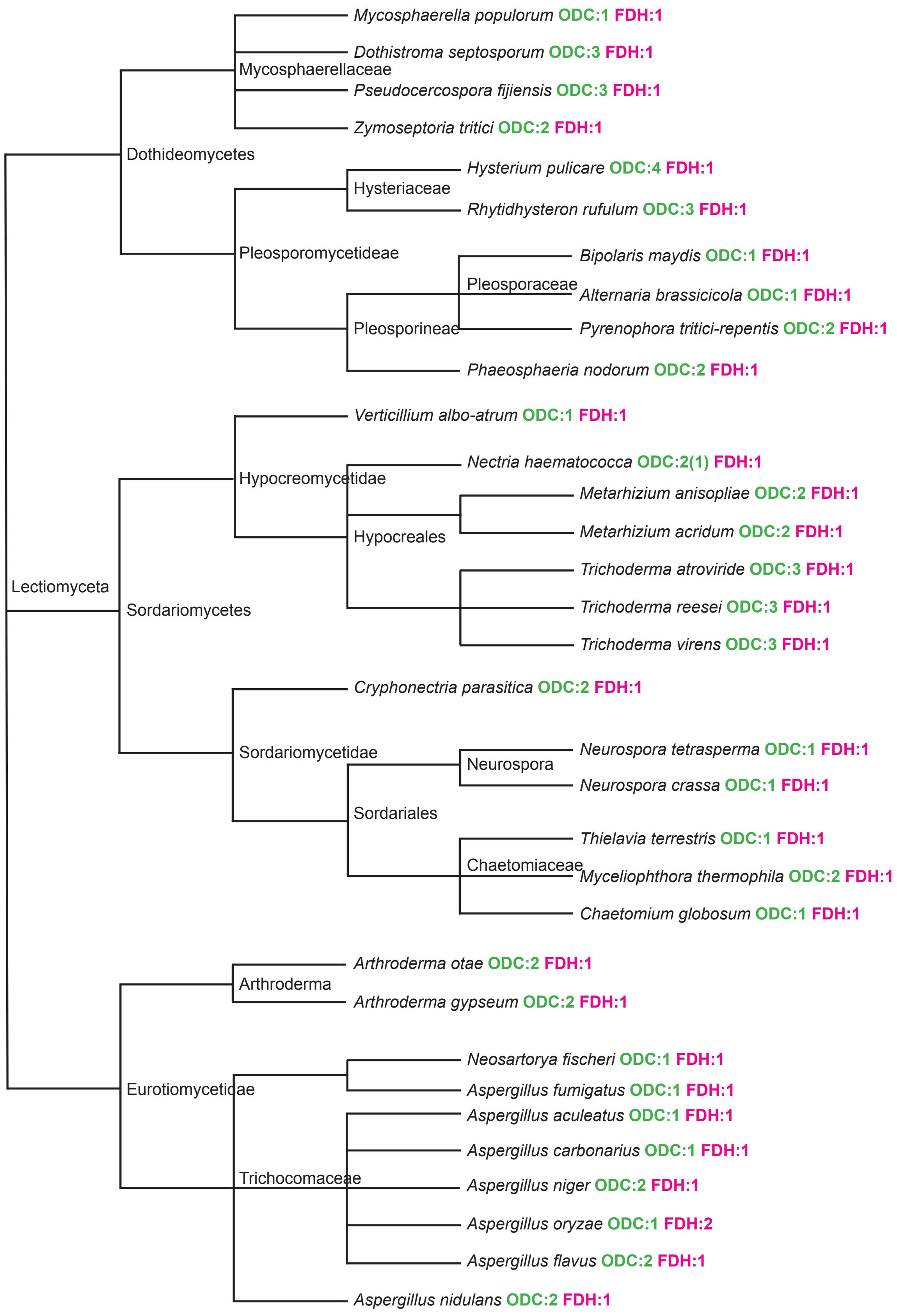

Mycosphaerella populorum ODC:1 FDH:1
Dothistroma septosporum ODC:3 FDH:1
Mycosphaerellaceae
Pseudocercospora fijiensis ODC:3 FDH:1
Zymoseptoria tritici ODC:2 FDH:1
Dothideomycetes
Hysterium pulicare ODC:4 FDH:1
Hysteriaceae
Rhytidhysteron rufulum ODC:3 FDH:1
Pleosporomycetideae
Bipolaris maydis ODC:1 FDH:1
Pleosporaceae
Alternaria brassicicola ODC:1 FDH:1
Pleosporineae
Pyrenophora tritici-repentis ODC:2 FDH:1
Phaeosphaeria nodorum ODC:2 FDH:1
Verticillium albo-atrum ODC:1 FDH:1
Nectria haematococca ODC:2(1) FDH:1
Hypocreomycetidae
Metarhizium anisopliae ODC:2 FDH:1
Hypocreales
Metarhizium acridum ODC:2 FDH:1
Trichoderma atroviride ODC:3 FDH:1
Lectiomyceta
Sordariomycetes
Trichoderma reesei ODC:3 FDH:1
Trichoderma virens ODC:3 FDH:1
Cryphonectria parasitica ODC:2 FDH:1
Sordariomycetidae
Neurospora tetrasperma ODC:1 FDH:1
Neurospora
Neurospora crassa ODC:1 FDH:1
Sordariales
Thielavia terrestris ODC:1 FDH:1
Chaetomiaceae
Myceliophthora thermophila ODC:2 FDH:1
Chaetomium globosum ODC:1 FDH:1
Arthroderma otae ODC:2 FDH:1
Arthroderma
Arthroderma gypseum ODC:2 FDH:1
Neosartorya fischeri ODC:1 FDH:1
Eurotiomycetidae
Aspergillus fumigatus ODC:1 FDH:1
Aspergillus aculeatus ODC:1 FDH:1
Aspergillus carbonarius ODC:1 FDH:1
Trichocomaceae
Aspergillus niger ODC:2 FDH:1
Aspergillus oryzae ODC:1 FDH:2
Aspergillus flavus ODC:2 FDH:1
Aspergillus nidulans ODC:2 FDH:1
