# Supplementary material for: Oxalate-Metabolising Genes of the White-Rot Fungus Dichomitus squalens Are Differentially Induced on Wood and at High Proton Concentration
Source: PLoS One. 2014 Feb 5;9(2):e87959. doi: 10.1371/journal.pone.0087959 (PMC3914892; doi:10.1371/journal.pone.0087959)
Supplement: Table S1 — List of species used in this study split in ascomycetes and basidiomycetes. Numbers of putative ODC and FDH encoding gene models are listed as are the number of gene models that have likely originated from recent gene duplication. (PDF) [file pone.0087959.s003.pdf]

Supplementary Table 1. List of species used in this study split in ascomycetes and basidiomycetes. Numbers of putative ODC and FDH encoding gene models are listed as are the number of gene models that have likely originated from recent gene duplication.

Basidiomycetes*	Synonyms	ODC	Recent duplication	FDH	Recent duplication
Agaricus bisporus		1	0	1	0
Auricularia delicata		3	1	1	0
Bjerkandera adusta		5	1	2	0
Ceriporiopsis subvermispora		7	3	1	0
Coniophora puteana		2	1	2	0
Coprinopsis cinerea		1	0	1	0
Cryptococcus neoformans		0	0	1	0
Dacryopinax sp.		2	1	2	1
Dichomitus squalens		5	0	3	0
Fomitiporia mediterranea		2	1	1	0
Fomitopsis pinicola		3	1	2	0
Ganoderma sp.		4	0	3	0
Gloeophyllum trabeum		3	0	2	0
Heterobasidion irregulare	*Heterobasidion annosum*	3	1	3	1
Laccaria bicolor		1	0	0	0
Malassezia globosa		0	0	1	0
Melampsora larici-populina		1	0	1	0
Phanerochaete chrysosporium		5	0	1	0
Phlebia brevispora		5	0	2	0
Phlebiopsis gigantea		5	0	2	0
Pleurotus ostreatus		2	0	1	0
Postia placenta		3	0	4	1
Punctularia strigosozonata		2	1	1	0
Rhodotorula graminis		0	0	1	0
Schizophyllum commune		5	2	1	0
Serpula lacrymans		2	0	2	0
Sporobolomyces roseus		0	0	1	0
Stereum hirsutum		2	1	2	0
Trametes versicolor		5	0	2	0
Ustilago maydis		0	0	1	0
Wolfiporia cocos		3	0	3	0

*current name of the species according to NCBI Taxonomy Browser (http://www.ncbi.nlm.nih.gov/Taxonomy/taxonomyhome.html/index.cgi)

Ascomycetes*	Synonyms	ODC	Recent duplication	FDH	Recent duplication
Aspergillus aculeatus		1	0	1	0
Alternaria brassicicola		1	0	1	0
Aspergillus carbonarius		1	0	1	0
Aspergillus flavus		2	0	1	0
Aspergillus fumigatus		1	0	1	0
Arthroderma gypseum		2	0	1	0
Aspergillus nidulans		2	0	1	0
Aspergillus niger		2	0	1	0
Aspergillus oryzae		1	0	2	0
Arthroderma otae		2	0	1	0
Bipolaris maydis	*Cochliobolus heterostrophus*	1	0	1	0
Chaetomium globosum		1	0	1	0
Cryphonectria parasitica		2	0	1	0
Dothistroma septosporum		3	0	1	0
Hysterium pulicare		4	0	1	0
Metarhizium acridum		2	0	1	0
Metarhizium anisopliae		2	0	1	0
Myceliophthora thermophila		2	0	1	0
Mycosphaerella populorum	*Septoria musiva*	1	0	1	0
Neurospora crassa		1	0	1	0
Neurospora discreta		0	0	1	0
Neosartorya fischeri		1	0	1	0
Nectria haematococca		2	1	1	0
Neurospora tetrasperma		1	0	1	0
Ogataea angusta	*Hansenula polymorpha*	0	0	1	0
Phaeosphaeria nodorum	*Stagonospora nodorum*	2	0	1	0
Pseudocercospora fijiensis	*Mycosphaerella fijiensis*	3	0	1	0
Pyrenophora tritici-repentis		2	0	1	0
Rhytidhysteron rufulum		3	0	1	0
Saccharomyces cerevisiae		0	0	1	0
Scheffersomyces stipitis		0	0	2	1
Trichoderma atroviride		3	0	1	0
Trichoderma reesei		3	0	1	0
Thielavia terrestris		1	0	1	0
Trichoderma virens		3	0	1	0
Verticillium albo-atrum		1	0	1	0
Zymoseptoria tritici	*Mycosphaerella graminicola*	2	0	1	0
